# Vocal Behavior of the Elusive Purple Frog of India (*Nasikabatrachus sahyadrensis*), a Fossorial Species Endemic to the Western Ghats

**DOI:** 10.1371/journal.pone.0084809

**Published:** 2014-02-07

**Authors:** Ashish Thomas, Robin Suyesh, S. D. Biju, Mark A. Bee

**Affiliations:** 1 Systematics Lab, Department of Environmental Studies, University of Delhi, Delhi, India; 2 Department of Ecology, Evolution and Behavior, University of Minnesota, St. Paul, Minnesota, United States of America; Queen Mary, University of London, United Kingdom

## Abstract

Quantitative descriptions of animal vocalizations can inform an understanding of their evolutionary functions, the mechanisms for their production and perception, and their potential utility in taxonomy, population monitoring, and conservation. The goal of this study was to provide the first acoustical and statistical analysis of the advertisement calls of *Nasikabatrachus sahyadrensis*. Commonly known as the Indian purple frog, *N. sahyadrensis* is an endangered species endemic to the Western Ghats of India. As the only known species in its family (Nasikabatrachidae), it has ancient evolutionary ties to frogs restricted to the Seychelles archipelago (Sooglossidae). The role of vocalizations in the behavior of this unique species poses interesting questions, as the animal is fossorial and potentially earless and it breeds explosively above the soil for only about two weeks a year. In this study, we quantified 19 acoustic properties of 208 calls recorded from 10 males. Vocalizations were organized into distinct call groups typically composed of two to six short (59 ms), pulsatile calls, each consisting of about five to seven pulses produced at a rate of about 106 pulses/s. The frequency content of the call consisted of a single dominant peak between 1200–1300 Hz and there was no frequency modulation. The patterns of variation within and among individuals were typical of those seen in other frogs. Few of the properties we measured were related to temperature, body size, or condition, though there was little variation in temperature. Field observations and recordings of captive individuals indicated that males engaged in both antiphonal calling and call overlap with nearby calling neighbors. We discuss our findings in relation to previous work on vocal behavior in other fossorial frogs and in sooglossid frogs.

## Introduction

Bioacoustic studies play fundamental roles in understanding and resolving several issues related to the study of anuran amphibians (frogs and toads). Given the importance of acoustic signaling in the breeding ecology of most frogs [1], [2], detailed acoustical and statistical descriptions of signals are an important first step toward understanding the reproductive and social behaviors of anurans [3]–[5]. Acoustic data are increasingly being used in combination with morphological and molecular data in integrative taxonomic studies of frogs [6]–[11]. Furthermore, basic knowledge of a species' acoustic behavior has important implications for conservation in at least two respects. On the one hand, bioacoustic data can be used as a noninvasive tool for the purposes of population census and monitoring. The integration of bioacoustics with other data sources can be important for effective conservation assessment, planning, and management, especially for threatened and endangered species [12]–[17]. On the other hand, detailed knowledge of species' acoustic behavior is necessary to assess the potential for anthropogenic noise to disrupt normal patterns of signal use and perception in ways that might interfere with reproduction and ultimately with population viability [18]–[22]. Given the global decline in amphibians [23], integrating bioacoustics with anuran conservation is an important goal.

**Figure 1:**
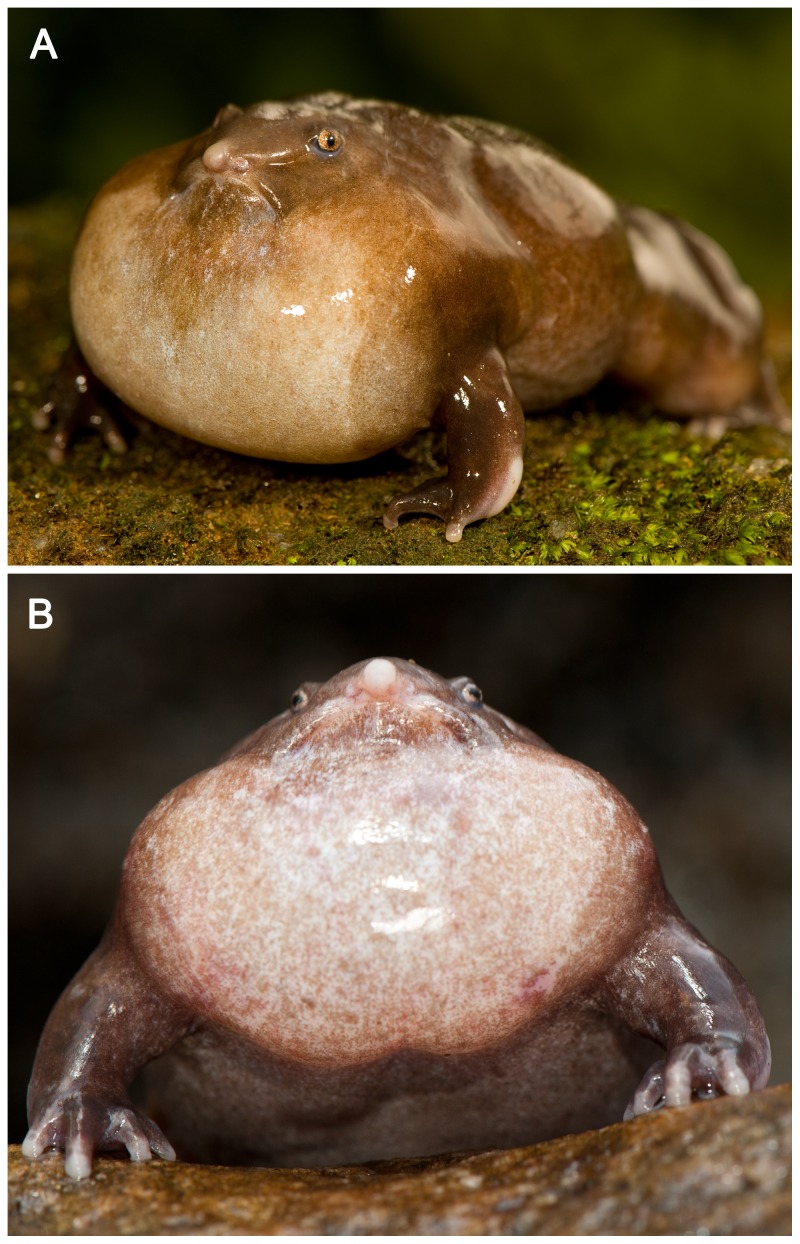
*Nasikabatrachus sahyadrensis* calling. Dorsolateral (a) and frontal (b) views of a calling male that was removed from under the soil at the entrance of the tunnel from which it had been calling. The male was induced to call above ground after brief exposure to a female.

The aim of the present study was to provide the first quantitative analysis of the vocalizations of an elusive and unique frog, *Nasikabatrachus sahyadrensis* [24]. An endangered species [25], *N. sahyadrensis* is endemic to the Western Ghats of India and is the only known species in the family Nasikabatrachidae [24], [26]. Phylogenetic analyses reveal that the family has very deep evolutionary roots, having diverged from its sister taxon, the Sooglossidae (a family of four diminutive species endemic to the Seychelles archipelago), before the origins of most other neobatrachian families [24], [27], [28]. With its dark, plump body and characteristic pointed snout (Figure 1) it has an appearance that is unlike that of most other frogs. Another morphological feature of notable importance for acoustic communication is the lack of an external tympanum [24], [29], suggesting the species may also lack a tympanic middle ear. The species also has an elusive lifestyle that makes it is a challenge to study, especially for bioacoustics. It is fossorial and an ‘explosive breeder’ [30], emerging from under the soil for only one to two weeks each year to breed. In our experience, males have only been observed to call from under a thin layer of soil near the opening of narrow tunnels filled with loose soil. Although recent research has shed some light on the natural history and larval development of the species [26], [31], information about the acoustic structure of its vocalizations remains limited [26].

Here, we provide a quantitative description of the advertisement call of *N. sahyadrensis*. We report measures of central tendency and dispersion for 19 acoustic properties measured for 208 advertisement calls recorded from 10 individuals. We also describe the patterns of variation in calls both within and among individuals, and evaluate the role of temperature, body size, and condition as potential sources of variation. Our study significantly extends a previous report by Zachariah et al. [26], who provided a brief and largely qualitative description of one call produced by one male in their study of reproduction and development. We discuss our results in light of previous work on other fossorial and earless frogs and the four related species of sooglossids from the Seychelles archipelago.

## Materials and Methods

### Ethics statement

This was an observational field study of free-ranging animals. All animals recorded as part of this study were collected for taking body size measurements and released unharmed at the location of the calling site where they were collected. Eight of 10 animals were released immediately after size measurements were taken, and the remaining two animals were released within 24 h of collection. The experimental protocol adhered to the Animal Behavior Society guidelines for the use of animals in research and was approved by the University of Minnesota Institutional Animal Care and Use Committee (#1202A10178).

### Study site

Calling males were recorded between 20 and 29 April 2012 in Methotti, Kulamaav (09°49′N, 76°53′E, 560 m above sea level), a tribal settlement area on the outskirts of the Idukki Wildlife Sanctuary, Kerala. The field site was on public land maintained by the Kerala Forest Department, which granted permission to SDB for conducting scientific research in forest areas (Kerala Forest Department, No. WL 12-1830/2009 and WL 10-2606/12). This site is located about 10 km from the type locality of the species [24]. The area experiences typical monsoon weather, with pre-monsoon showers during the months of April and May followed by the monsoon season that begins towards the end of May or beginning of June. Calling and breeding activity take place during pre-monsoon rains. The drainage system in the study area consists of first and second order seasonal streams that drain into the Muvattupuzha River basin. We worked along hillsides near one of these seasonal streams where we had heard vocalizing males during visits to the area in previous years. We measured the distance between each recorded male's calling site and the nearest stream bank using a metered rope. On one prior visit to the nearby type locality for this species (Kattapana, Doublecut, 09°45′N, 77°05′E, 900 m above sea level) in April 2004, we had determined the calling locations of 15 different males in a 75 m×100 m area and measured the distance to each male's nearest calling neighbor; we report these data here as well.

### Acoustic recordings

Recordings were made between 1700 and 0100 h, typically just after a heavy downpour when males called most actively. Recordings of 10 individual males (16-bit, 44.1 kHz) were made onto a Fostex FR2LE solid-state recorder using a handheld Sennheiser MKH 416 microphone mounted in a Rycote WS4 blimp windscreen. The recording tip of the microphone was positioned approximately 30–50 cm from the area of the soil from which the focal animal was calling. The gain setting of the recorder was adjusted prior to the onset of each recording and remained constant during a recording.

At the completion of each recording, we captured the male by rapidly digging with a spade into the soil under which the animal had been calling. In response to this disturbance, males quickly began retreating deeper into the soil. Consequently, though males called from just below the soil surface, they were typically captured at depths of about 20–30 cm (Table 1). Immediately after the animal was captured, we used a Jennson Delux thermometer (±0.2°C) to record the dry-bulb and wet-bulb air temperatures just above the soil where the animal had been calling, as well as the temperature of the loose soil at the animal's calling position. While we recognize recording soil temperature at a male's calling site prior to disturbing the soil would have been ideal, this was not possible in practice, as males retreated deeper underground in response to any disturbance by us of its calling site. As reported in Table 1, mean air and soil temperatures were similar (within 0.8°C), and there was very little variation in temperature across the 10 sound recordings (≤1.6°C). We used dial calipers to measure each male's snout-to-vent length (SVL, to the nearest 0.1 mm) and a portable, electronic balance to measure body mass (to the nearest 0.1 g). These two measures of body size were used to compute an index of body condition (i.e. length-independent mass) following Baker [32]. The condition index was estimated as the residuals from a regression of the cube root of mass on SVL divided by SVL. Descriptive statistics for measures of body size and condition are reported in Table 1. After measurements were taken, we replaced any displaced soil and released the animal into the loose soil from which it was collected. On subsequent days, animals were usually observed calling from these same locations or in close proximity to their original calling sites.

**Table 1:** Descriptive statistics for various environmental, phenotypic, and physical properties.

Property	*X¯*	SD	Minimum	Maximum
Depth below soil at which male captured (cm)	26.5	10.9	15.0	50.0
Dry-bulb air temperature (°C)	22.8	0.5	21.8	23.2
Wet-bulb air temperature (°C)	22.1	0.5	21.4	22.5
Soil temperature (°C)	22.9	0.5	21.5	23.1
Snout-vent-length (SVL, mm)	61.3	2.3	58.0	65.0
Mass (g)	28.7	2.2	23.5	31.0
Condition index (×10^3^)	0.0	0.7	−1.7	0.8
Distance calling from stream (m)	14.0	7.5	5.0	26.0
Distance between calling sites (m)	10.8	9.6	0.6	27.0

During the field portion of our study, we periodically heard two males calling in close proximity to one another engage in what appeared to be vocal interactions. These interactions consisted of repeated and alternating bouts of call overlap and call alternation. Unfortunately, we were unable to capture any of these natural interactions in our sound recordings. We noticed, however, that males captured and held in captivity as part of another ongoing study would not only vocalize, but also engage in very similar vocal interactions with other males also held in captivity. Therefore, simply to illustrate the type of vocal interactions that can occur in this species, we recorded one such interaction between two captive males that were temporarily housed in a large plastic tub after their collection. The two animals were calling within about 50 cm of each other during the recording.

### Acoustical analyses

**Figure 2:**
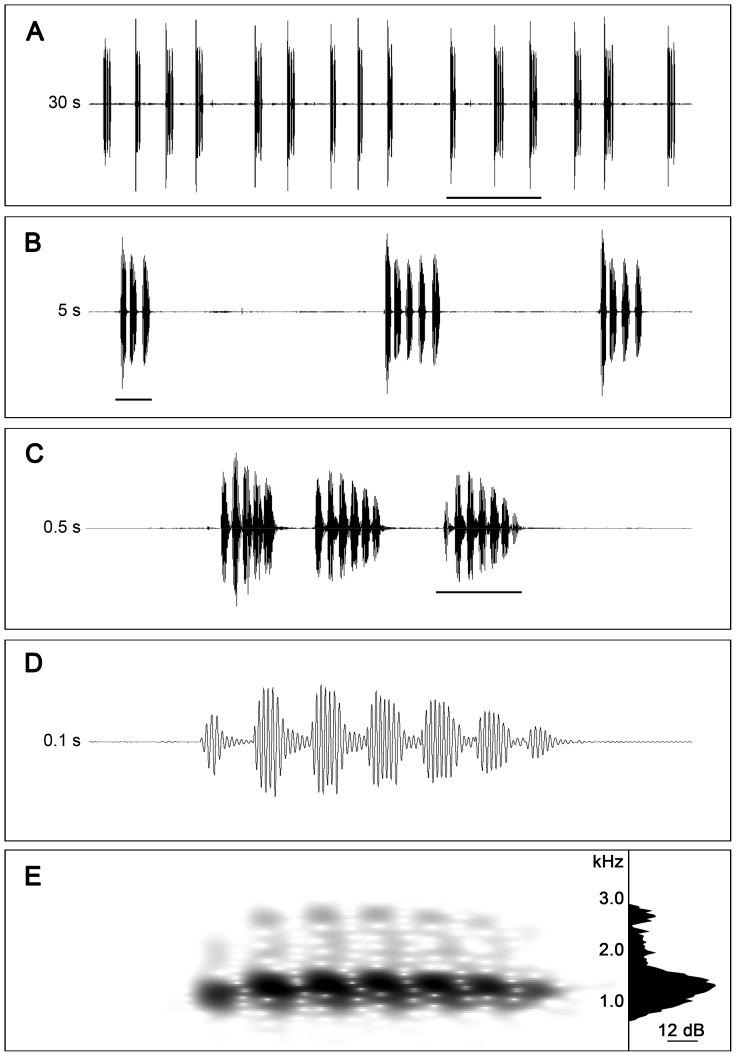
Advertisement calls of a male *Nasikabatrachus sahyadrensis*. (a) 30-s segment of continuous, spontaneous calling by a single male. (b) 5-s segment showing the three consecutive call groups underlined in (a). (c) 0.5-s segment showing the three calls of the call group underlined in (b). (d) 0.1 s segment showing the call underlined in (c). (e) Spectrogram of the call illustrated in (d); *Inset*: power spectrum averaged over the duration of the call depicted in (d). The entire 30-s segment depicted in this figure is included as an audio file in the Supporting Information for this article (Audio S1).

Informally observing and listening to the calling of *N. sahyadrensis* revealed that males produced pulsatile calls organized into short call groups comprising several calls each (Figure 2). Call groups themselves were repeated every couple of seconds. Therefore, we based our analyses of various acoustic properties on this hierarchically organized temporal structure. In the Supporting Information for this article, we include the sound clip depicted in Figure 2 (Audio S1) and a short video clip (Video S1) of a different calling male. The video was made using a Sony HDR-XR520V video camera (60 frames/s). Prior to video recording, the male was captured, removed from beneath the soil, and induced to call on the soil surface using brief exposure to a female. The calls this male produced were acoustically similar to the calls produced when males called from beneath the soil surface (cf. Audio S1 and Video S1).

Table 2 provides detailed descriptions of the acoustic properties measured using Raven Pro v1.4, and readers are referred to this table for detailed descriptions. Briefly, we measured between 3 and 10 (median = 5.5) complete call groups per frog such that a minimum of 20 consecutive calls per frog was analyzed (range = 20 to 23 calls per frog, 208 calls total). For call groups, we determined the number of calls in the group, the duration of the call group, and the duration of the interval between consecutive call groups (Table 2). For calls, we measured several temporal properties that included pulse rate, the duration of the period of the first, middle, and next-to-last pulses in the call, the number of pulses per call, call duration, the call's amplitude envelope (rise time, fall time, and the peak power of the first and last pulses relative to the middle pulse), and the interval between consecutive calls within a call group (Table 2). We also measured one spectral property by determining the frequency of greatest relative amplitude (hereafter, ‘dominant frequency’) after averaging the spectrum over an entire call. There was no indication that frequency modulation occurred during a call. Finally, for each analyzed call, we selected the middle pulse (which was usually the pulse of greatest relative amplitude) and measured its duration as well as four properties describing the pulse's amplitude envelope that included the rise and fall times and the times between onset or offset and the points of 50% amplitude (Table 2). For calls with an even number of pulses we selected as the middle pulse that pulse marking the beginning of the second half of the call (i.e. pulse number k/2+1, where *k* is the total number of pulses). The signal-to-noise ratios of our recordings were sufficiently high to unambiguously determine the onsets and offsets of individual sound elements.

**Table 2:** Descriptions of the acoustic properties measured for call groups, calls, and pulses.

Acoustic unit	Property	Description
Call groups	Calls per call group	Count of the number of calls in a call group.
	Call group duration (ms)	Duration of a call group measured from the onset of the first pulse in the first call to the offset of the last pulse in the last call.
	Inter-call-group interval (ms)	Duration of the interval between consecutive call groups measured from the offset of the last pulse of the last call in one call group to the onset of the first pulse of the first call in the next consecutive call group.
Calls	Pulses per call	Count of the number of pulses in a call (*k*).
	Pulse rate (pulses/s)	Number of pulses per call (*k*) minus 1 (i.e. *k* – 1), divided by time between the onset of the first pulse and onset of the last pulse.
	Pulse period (ms)	Time between the onset of one pulse and the onset of the next consecutive pulse, measured separately for the first (*k* = 1), middle (k/2+1), and next-to-last (*k*-1) pulses of a call.
	Call duration (ms)	Time between the onset of the first pulse of a call and the offset of the last pulse of the call.
	Inter-call interval (ms)	Duration of the interval between consecutive calls within a call group measured from the offset of the last pulse of one call to the onset of the first pulse of the next consecutive call in the call group.
	Call rise time (ms)	Time between the onset of the first pulse of a call and the point of peak amplitude in the pulse of greatest amplitude.
	Call fall time (ms)	Time between the point of peak amplitude in the pulse of greatest amplitude and the offset of the last pulse of a call.
	Relative peak power (dB)	Peak power of the first and last pulse of a call measured relative to the middle pulse of the call (0 dB).
	Dominant frequency (Hz)	Frequency of maximum amplitude measured from a power spectrum generated using Raven's selection spectrum function over the duration of the entire call (FFT size = 1024 pts, Hanning window, 43.1 Hz resolution).
Pulses†	Pulse duration (ms)	Time between the onset and the offset of the middle pulse in a call.
	Pulse rise time (ms)	Time between the onset and the point of maximum amplitude of the middle pulse in a call.
	Pulse 50% rise time (ms)	Time between the onset and the point of 50% maximum amplitude of the middle pulse in a call.
	Pulse fall time (ms)	Time between the point of maximum amplitude and the offset of the middle pulse in a call.
	Pulse 50% fall time (ms)	Time between the point of 50% maximum amplitude and the offset of the middle pulse in a call.

† Values for pulses were determined for the middle pulse (k/2+1), where *k* is the total number of pulses). Because there was no silent interval between pulses, pulse duration is equivalent to the period of the middle pulse.

### Statistical analyses

We used MATLAB v7.12.0 to compute descriptive statistics for all measured acoustic properties, including the arithmetic mean (*X¯*), standard deviation (SD), and the minimum and maximum values. For properties that constituted counts (i.e. calls per call group and pulses per call), we report medians and interquartile ranges in lieu of the *X¯* and SD. We report the absolute minimum and maximum based on all recorded calls (*N* = 208) as well as the mean, SD and range of individual means based on first averaging the calls of each individual (*N* = 10). We report coefficients of variation (as percentages, CV = 100%×SD/*X¯*) computed two different ways. First, we computed the among-individual coefficient of variation (CV_a_) using the SD and *X¯* computed based on 10 individual mean values. Second, we computed a within-individual coefficient of variation (CV_w_) for each of the 10 individuals using the SD and *X¯* based on the calls recorded from that individual. Both CV_a_ and CV_w_ have been used extensively to describe patterns of individual variation in the acoustic properties of anuran and insect calls [1], [33].

Because anurans are ectotherms, the temporal properties of their vocalizations are frequently related to temperature [1]. Hence, quantitative analyses of anuran calls demand consideration of the *potential* for variation in temperature at the time recordings are made to introduce variation in call properties. Spectral properties are often related to body size due to biophysical constraints of sound production [34]. Given the energetic demands of calling [2], there is also potential for call properties to be related to aspects of physical condition. We assessed the relationships between each acoustic property and temperature, body size, and condition using Spearman rank correlations. We used Friedman's tests to compare pulse rates across the calls within a call group and pulse periods across the first, middle, and next-to-last pulses within a call.

## Results

### Calling sites

Most commonly, males were heard calling from just below ground after episodes of rain, under a thin (e.g. 2–4 cm) layer of loose soil and humus. In some instances, we observed movement of the soil that was coincident with vocalizations. Upon closer inspection, and after clearing away leaf litter and removing the thin layer of soil beneath which males called, we exposed the opening of small tunnels (e.g. 2–5 cm in diameter) partially filled with an accumulation of loose soil. The slightest disturbance in the vicinity of a male's calling site usually caused males to cease calling and immediately retreat deeper below the surface of the ground. On average, male calling sites were located at distances of about 14 m from the seasonal stream and they were spaced about 11 m apart (Table 1).

### Call groups

Advertisement calls were organized into distinct call groups that were repeated in rapid sequence and typically comprised between two and six calls each (Table 3). Figure 2 illustrates a series of consecutive call groups (Figure 2a) produced by one individual, as well as a detail of three consecutive call groups (Figure 2b) consisting of three (Figure 2c), five, and four calls, respectively. Most call groups were between 150 and 700 ms in duration and were repeated after intervals ranging from about 1 to 4 s in duration.

**Table 3:** Descriptive statistics for call groups, calls, and pulses, including means (*X¯*), standard deviations (SDs), ranges, and coefficients of variation.

		Absolute range		Data based on individual means								
		(*N* = 208 calls)		(*N* = 10 individuals)								
Acoustic unit	Property	Min	Max	*X¯*	SD	Min	Max	CV_a_	CV_w_ (*X¯*)	CV_w_ (Min)	CV_w_ (Max)	CV_a_∶CV_w_
Call groups	Calls per call group†	2.0	9.0	3.5	2.0	2.0	6.0	32.2	13.7	0.0	31.2	2.4
	Call group duration (ms)	136.7	881.7	380.3	145.6	155.4	652.8	38.3	17.8	0.9	30.6	2.1
	Inter-call-group interval (ms)	875.9	14974.7	1684.2	867.3	1183.6	4035.4	51.5	34.2	11.8	113.4	1.5
Calls	Pulses per call (*k*)†	5.0	8.0	6.0	0.5	5.5	7.0	4.9	10.9	7.8	15.4	0.4
	Pulse rate (pulses/s)	95.8	118.2	105.6	3.2	100.9	110.1	3.0	3.9	2.9	4.6	0.8
	Pulse period (ms) – first pulse	6.4	11.6	9.8	0.5	9.1	10.5	5.2	8.6	5.5	13.5	0.6
	Pulse period (ms) – middle pulse	7.3	10.3	9.3	0.2	9.1	9.7	2.0	4.1	2.1	6.7	0.5
	Pulse period (ms) – *k*-1 pulse	7.3	14.7	9.2	0.4	8.6	9.7	4.2	6.6	3.0	14.9	0.6
	Call duration (ms)	44.2	77.3	59.0	3.6	52.7	63.7	6.1	10.5	7.5	15.8	0.6
	Inter-call interval (ms)	11.8	78.3	42.5	7.3	24.7	51.7	17.2	31.1	22.3	48.6	0.6
	Call rise time (ms)	3.5	44.3	19.5	3.0	15.9	25.0	15.2	27.5	17.2	47.2	0.6
	Call fall time (ms)	7.5	64.7	39.5	4.4	32.4	47.8	11.2	18.3	10.2	26.6	0.6
	Relative peak power (dB) – first pulse	−26.6	2.0	−6.4	1.7	−9.3	−3.9	26.5	91.1	135.0	61.4	0.3
	Relative peak power (dB) – last pulse	−20.7	3.2	−6.7	1.8	−9.5	−4.9	26.6	61.8	86.0	36.4	0.4
	Dominant frequency (Hz)	1205.9	1378.1	1230.5	44.4	1205.9	1315.5	3.6	1.9	0.0	6.6	1.9
Pulses	Pulse duration (ms)	7.3	10.3	9.3	0.2	9.1	9.7	2.0	4.1	2.1	6.7	0.5
	Pulse rise time (ms)	1.5	5.1	2.7	0.6	1.8	3.7	23.8	18.2	7.2	29.8	1.3
	Pulse 50% rise time (ms)	0.6	1.9	1.0	0.2	0.8	1.3	16.4	14.6	6.2	23.4	1.1
	Pulse fall time (ms)	4.2	8.2	6.6	0.5	6.0	7.3	7.9	9.7	5.3	17.2	0.8
	Pulse 50% fall time (ms)	1.7	5.5	3.9	0.5	3.1	4.7	13.1	14.9	10.6	20.5	0.9

† For calls per call group and pulses per call, the values reported in the columns headed *X¯* and SD, respectively, are the median and interquartile range. Note, however, that coefficients of variation for these two properties are computed from the *X¯* and SD.

As revealed by considering the values of CV_a_ (32.2% to 51.5%) and CV_w_ (13.7% to 34.2%) for call group properties (Table 3), the temporal organization of call groups was more variable among individuals than within individuals. This was reflected in CV_a_∶CV_w_ ratios that ranged between 1.5 and 2.4 (Table 3). The CV_a_∶CV_w_ ratios for the number of calls per call group (2.4) and call group duration (2.1) were higher than for any other acoustic property measured, and that for inter-call-group interval (1.5) was higher than all remaining acoustic properties with the exception of dominant frequency.

There were no significant correlations between any properties of call groups and temperature, SVL, or mass (Table 4). The number of calls per call group was negatively related to body condition (*r_s_* = −0.69, *P* = 0.03; see Figure S1), but not significantly so after a Bonferroni correction for multiple comparisons. No other call group properties were correlated with condition.

**Table 4:** Results of Spearman rank correlations between acoustic properties and temperature, body size, and condition (*N* = 10).

		Dry-bulb		Wet-bulb		Soil							
		air temperature		air temperature		temperature		SVL		Mass		Condition	
Acoustic unit	Property	*r_s_*	*P*	*r_s_*	*P*	*r_s_*	*P*	*r_s_*	*P*	*r_s_*	*P*	*r_s_*	*P*
Call groups	Calls per call group	0.14	0.70	0.35	0.32	0.16	0.66	−0.05	0.89	−0.22	0.54	−0.69†	0.03
	Call group duration (ms)	0.15	0.67	0.42	0.23	0.15	0.68	−0.12	0.76	−0.30	0.41	−0.59	0.08
	Inter-call-group interval (ms)	−0.30	0.41	−0.20	0.58	−0.18	0.62	−0.58	0.09	−0.45	0.19	0.21	0.56
Calls	Pulses per call (*k*)	0.34	0.34	0.23	0.52	0.53	0.11	0.05	0.90	0.09	0.81	0.27	0.46
	Pulse rate (pulses/s)	−0.39	0.27	−0.52	0.13	0.06	0.87	0.54	0.11	0.52	0.13	0.05	0.89
	Pulse period (ms) – first pulse	0.15	0.68	0.23	0.53	0.02	0.95	−0.53	0.12	−0.50	0.14	−0.05	0.89
	Pulse period (ms) – middle pulse	0.35	0.33	0.37	0.29	−0.17	0.63	−0.44	0.20	−0.35	0.33	−0.01	1.00
	Pulse period (ms) – *k*-1 pulse	0.23	0.51	0.11	0.76	0.01	0.98	−0.61	0.07	−0.48	0.17	0.14	0.71
	Call duration (ms)	0.31	0.39	0.20	0.58	0.34	0.34	−0.22	0.54	−0.15	0.68	0.52	0.13
	Inter-call interval (ms)	0.15	0.68	0.48	0.16	−0.37	0.30	−0.26	0.47	−0.37	0.30	−0.37	0.30
	Call rise time (ms)	−0.25	0.48	0.30	0.40	−0.25	0.48	−0.26	0.47	−0.44	0.20	−0.52	0.13
	Call fall time (ms)	0.48	0.16	0.15	0.68	0.48	0.16	0.04	0.92	0.10	0.79	0.55	0.10
	Relative peak power (dB) – first pulse	−0.27	0.46	−0.44	0.20	0.13	0.73	0.03	0.95	−0.01	1.00	0.15	0.68
	Relative peak power (dB) – last pulse	0.25	0.48	0.15	0.68	0.34	0.34	−0.42	0.23	−0.48	0.17	0.19	0.61
	Dominant frequency (Hz)	0.09	0.81	−0.41	0.23	0.74†	0.01	0.14	0.71	0.16	0.65	0.38	0.28
Pulses	Pulse duration (ms)	0.35	0.33	0.37	0.29	−0.17	0.63	−0.44	0.20	−0.35	0.33	−0.01	1.00
	Pulse rise time (ms)	0.11	0.76	0.21	0.56	−0.44	0.20	−0.65†	0.05	−0.58	0.09	−0.07	0.86
	Pulse 50% rise time (ms)	−0.43	0.21	−0.14	0.69	−0.75†	0.01	−0.53	0.12	−0.44	0.20	−0.12	0.76
	Pulse fall time (ms)	0.02	0.95	−0.14	0.69	0.51	0.13	0.66†	0.04	0.62	0.06	−0.01	1.00
	Pulse 50% fall time (ms)	−0.76†	0.01	−0.35	0.33	−0.48	0.16	−0.31	0.39	−0.38	0.28	−0.25	0.49

† Scatterplots depicting correlations with *P*-values below the conventional (uncorrected) α level of 0.05 are included in Figure S1.

### Calls

A typical advertisement call is illustrated in Figure 2d. Advertisement calls had a pulsatile temporal structure and consisted of about 5 to 7 pulses each. Pulses were typically produced at a rate of about 100 to 111 pulses/s (Table 3). Pulse rates tended to be faster in the first call in a call group (*X¯* = 111.6 pulses/s; *N* = 10 individuals) compared with subsequent calls in the group, which were similar (e.g. 102.0<*X¯*<103.5 pulses/s for calls two through six in a call group; *N* = 2 to 10 individuals). These differences were significant in a comparison of the first three calls within call groups, for which we had data for all 10 subjects (Friedman's test: *χ^2^* = 15, df = 2, *P*<0.01, *N* = 10 individuals). There was also a tendency for pulse rate to increase slightly over the duration of a single call, as revealed by significant differences in pulse period (which is reciprocally related to pulse rate) between the first, middle, and next-to-last pulses in the call (Table 3; Friedman's test: *χ^2^* = 7.4, df = 2, *P* = 0.02, *N* = 10 individuals per position).

Consisting of relatively few pulses produced at fast pulse rates, advertisement calls were consequently short, typically ranging between 52 and 64 ms in duration (Table 3). The calls within a call group were produced in rapid succession and separated by short inter-call intervals lasting about 24 to 52 ms in duration (Figure 2c; Table 3). The amplitude envelope of the call was characterized by a rise time (*X¯* = 19.5 ms) that was about half the duration of the fall time (*X¯* = 39.5 ms). The first and the last pulse of the call were produced at amplitudes that averaged −6.4 dB and −6.7 dB, respectively, relative to the middle pulse (0 dB). Averaged over the duration of a call, the spectrum was characterized by a single, broad peak with a mean dominant frequency of 1230.5 Hz (Figure 2e). This peak was typically at least 20 to 30 dB higher in amplitude than any other spectral peaks in the call.

At the times recordings were made, we were struck by how similar the calls of the males we recorded sounded to each other. This subjective impression was borne out by objective analyses of coefficients of variation. All of the call properties we measured varied more within individuals than among individuals (i.e. CV_w_>CV_a_), yielding CV_a_∶CV_w_ ratios that were uniformly 0.6 or less (Table 3). Compared with call and pulse amplitude envelopes and inter-call interval, coefficients of variation were smaller for properties related to pulse rate (e.g. pulse rate, pulse duration, and pulse period), the duration of calls (e.g. call duration and pulse number), and the dominant frequency (Table 3).

In general, call properties were not related to temperature, body size, or condition (Table 4). The single exception was dominant frequency, which was positively related to soil temperature (*r_s_* = 0.74, *P* = 0.01), though not significantly so after a Bonferroni correction for multiple comparisons. Closer inspection of the data for dominant frequency suggested this relationship was spurious and resulted because one male with a low dominant frequency was recorded at the lowest soil temperature (21.5°C), whereas all other males were recorded at soil temperatures near 23.0°C (see Figure S1).

### Pulses

The individual pulses composing a call were about 9 ms in duration. Pulses had a short rise time (*X¯* = 2.7 ms), with the pulse reaching 50% of its maximum amplitude in the first 1 ms, on average. The pulse fall time (*X¯*  = 6.6 ms) was nearly 2.5× longer than the rise time. The offset of the pulse approximated an inverse exponential function, with the pulse decreasing to 50% of its maximum amplitude nearly 4 ms before the end of the pulse (Table 3).

Compared with call groups and calls, pulses exhibited similar magnitudes, but less coherent patterns, of variation within and among individuals (Table 3). While pulse duration and pulse fall times were more variable within individuals (i.e. CV_w_>CV_a_ and CV_a_∶CV_w_<1.0), the opposite was true for pulse rise times. With the exception of pulse duration, properties of the pulse amplitude envelope were similarly variable within and among individuals, with CV_a_∶CV_w_ ratios near 1.0 (0.8<CV_a_∶CV_w_<1.3).

Pulse duration was unrelated to temperature, body size, and condition (Table 4). Likewise, there was little indication that most properties of the pulse amplitude envelope were related to temperature, body size, and condition (Table 4). There were statistical trends indicating that pulse 50% rise time and 50% fall time were inversely related with soil temperature and dry-bulb air temperature (Table 4). However, in neither case were correlations significant following a Bonferroni correction for multiple comparisons. In addition, visual inspection of the data did not suggest strong relationships that would have warranted statistical corrections for temperature effects over the narrow range of temperatures we recorded (see Figure S1). There was some indication that pulse rise time and fall time, respectively, were inversely (*r_s_* = −0.65, *P*<0.05) and directly (*r_s_* = 0.66, *P* = 0.04) related to SVL (Table 4). Neither of these correlations was significant after a Bonferroni correction for multiple comparisons (see Figure S1).

### Calling interaction

**Figure 3:**
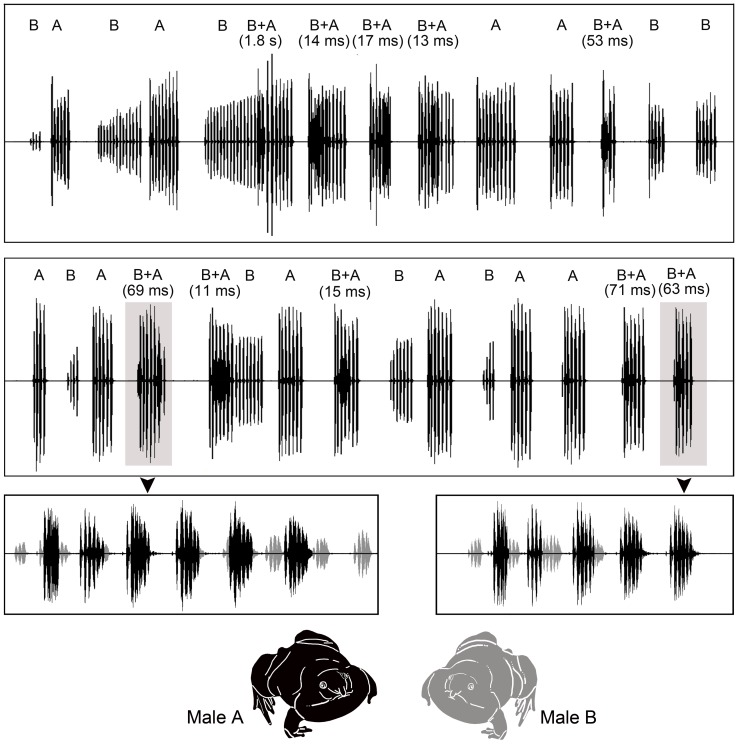
Vocal interaction in *Nasikabatrachus sahyadrensis*. Shown are two 23-s segments of a vocal interaction that occurred between two males (A and B) while held in close proximity in captivity. The letter above each series of calls corresponds to the male that produced the call. In instances of call overlap, the first letter indicates the male that called first and was subsequently overlapped by the male indicated by the second letter. Times correspond to the time between the onset of the overlapped male's call and the onset of the subsequent overlapping call. *Inset*: Shown here are details of the two shaded regions with the calls of male A and male B illustrated in different colors. The first and second 23-s segments of this interaction are included as audio files in the Supporting Information for this article (Audio S2 and Audio S3, respectively).

Figure 3 illustrates two 23-s segments from a vocal interaction between two males (A and B) that began calling while held in captivity. Our recordings of these two interactions are included in the Supporting Information (Audio S2 and Audio S3). As illustrated by these two examples, males frequently switched between alternating calls (A or B) and overlapping calls (B+A). In this particular interaction, every occurrence of call overlap involved male A overlapping the calls of male B. With only one exception, male A produced overlapping calls within 71 ms of the onset of male B's call (Figure 3). In several instances, male A's overlapping call began with a latency of just 11 to 17 ms relative to the onset of male B's call, which is slightly longer than the average period for first pulses (*X¯* = 9.8 ms). Hence, in these instances, the first pulse of male A's call was nearly synchronized to the second pulse of male B's call.

## Discussion

During the course of the present study, we heard and recorded only one type of vocalization produced by males of *N. sahyadrensis*. The call was short, pulsatile, and organized into rapidly repeated call groups usually composed of two to six calls each. Similar hierarchical organizations have been described in other anurans with calls of varying complexity [35]–[38]. This call was produced spontaneously by isolated males, as well as during vocal interactions with other males calling in close proximity. We take this vocalization to be the species' ‘advertisement call’ [30]. Whether the call has the same primary functions as advertisement calls in most other anurans [1], [2], namely mate attraction and male-male communication, remains to be determined empirically in future playback tests and behavioral studies. We observed neither females approaching calling males nor overt physical aggression among calling males during the course of our study.

Two common sources of variation in anuran vocalizations are temperature and body size [1]. Variation in temperature at the time recordings are made can introduce variation in calls and obscure estimates of the real population parameters (e.g. mean and variance) of call properties, especially temporal properties. Therefore, examining the *potential* for temperature effects is essential in all bioacoustical analyses of frog calls. In the present study, none of the call group, call, and pulse properties we measured were significantly correlated with temperature after correcting for multiple comparisons, and only dominant frequency, and pulse 50% rise and 50% fall times were correlated with temperature prior to this correction. The lack of any strong effect of temperature is perhaps not surprising given the very narrow range (≤1.6°C) of temperatures encountered during the study. Given this narrow temperature range, and the lack of any clear patterns of call variation due to variation in temperature, we elected not to statistically correct calls for temperature variation in our analyses. We believe recordings over a wider range of temperatures would be necessary to make meaningful corrections in this species.

Both within and among species, spectral call properties (e.g. dominant frequency) are often inversely related to male body size due to the biophysical constraints of sound production [1], [2]. However, in the present study, dominant frequency was unrelated to either SVL or mass. In fact, none of the call group, call, and pulse properties we measured was significantly related to body size after corrections for multiple comparisons. The only trends indicating possible size effects were observed for pulse rise and fall times. Larger males tended to produce pulses with relatively shorter onsets and longer offsets. There was also a non-significant trend for the males scoring lower on our condition index to produce call groups having more calls. None of these trends was significant after corrections for multiple comparisons.

The patterns of individual variation, indicated by estimates of CV_a_ and CV_w_, were similar to those reported for other frogs [1]. For example, dominant frequency was associated with the lowest CV_w_ of any property and had one of the highest CV_a_∶CV_w_ ratios, similar to reports for several other anuran species [3], [33], [36], [39]–[42]. Pulse rate and related properties (pulse period and pulse duration) were also stereotyped within individuals (e.g. CV_w_≤8.6%), and these properties also exhibited little variation among individuals (CV_a_≤5.2%), a general pattern also reported for several hylid treefrogs with pulsatile calls [33], [43]. Overall, there was a tendency for properties that varied most within individuals to also vary more among individuals (*r_s_* = 0.82, *P*<0.01; *N* = 19 after excluding pulse duration, which was equal to the period of the middle pulse). The most individually distinctive properties, that is, those with the highest CV_a_∶CV_w_ ratios, were related to the temporal structure of call groups, indicating that call group production differed among males, but was relatively stereotyped within males.

The single vocal interaction we recorded between two captive males was characterized by what appeared to be short, alternating bouts of antiphonal calling and extensive call overlap. Both behaviors are known to occur in male frogs engaged in close-range interactions with neighboring males in choruses [1], [2], [44]. Unfortunately, our recording of the interaction between these two males was too brief to establish statistically whether either male altered its regular calling period during the interaction. Hence, it remains possible that the alternation and overlap depicted in Figure 3 is more apparent than real. Nevertheless, the short latencies of most overlapping calls and the unidirectional nature of the overlap (i.e. A always overlapping B) are at least suggestive that call overlap might be a functionally important form of communication among neighboring males in this species. Playback experiments [45] or detailed measurements of the call timing patterns among multiple neighboring males, for example using a microphone array [46], will be required to test this hypothesis.

**Figure 4:**
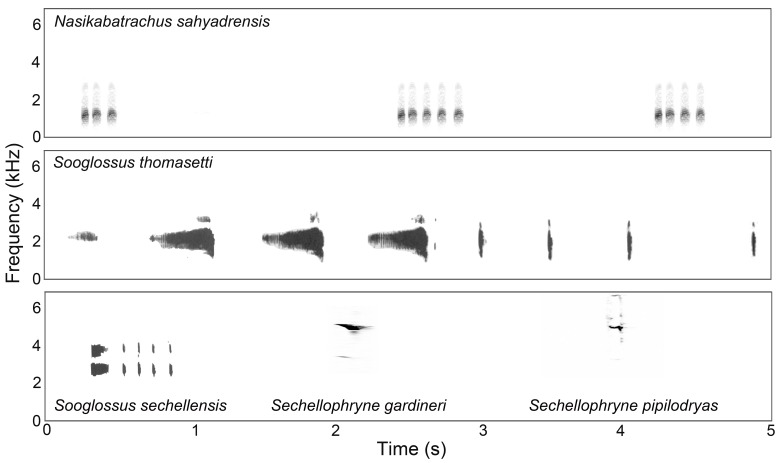
Comparison of vocalizations in Nasikabatrachidae and Sooglossidae. Shown here are spectrograms depicting (top) three call groups from *Nasikabatrachus sahyadrensis*; (middle) one call of *Sooglossus thomasetti*; and (bottom) one call each from *Sooglossus sechellensis*, *Sechellophryne gardineri*, and Sechellophryne pipilodryas. The calls of *Sooglossus thomasetti* and *Sooglossus sechellensis* reprinted from [48] under a CC BY license, with permission from the *Journal of Herpetology*, original copyright [1982, Society for the Study of Amphibians and Reptiles]. The calls of *Sechellophryne gardineri* and *Sechellophryne pipilodryas* were provided courtesy Dr. Justin Gerlach.

*Nasikabatrachus sahyadrensis* is the only known species from the family Nasikabatrachidae [24], [47]. The species is most closely related to the Sooglossidae, which comprises four species geographically restricted to the Seychelles archipelago in the Indian Ocean. The calls of these four sooglossid species have been described previously [48]–[50]. As illustrated in Figure 4, none of these calls bear much resemblance to the call of *N. sahyadrensis* aside from a pulsatile structure in two of the four species. In both *Sooglossus* (formerly *Nesomantis*) *thomasetti* and *Sooglossus sechellensis*, advertisement calls are composed of two distinctly different note types that are pulsed (Figure 4). In *S. thomasetti*, males commonly produce calls consisting of three to seven primary notes followed immediately by one to six secondary notes, whereas males of *S. sechellensis* produce calls having a single primary note followed by one to seven secondary notes [48]. Both of these species' calls are quite distinct from the calls of the other two closely related species within the family Sooglossidae, *Sechellophryne* (formerly *Sooglossus*) *gardineri* [48], [50] and *Sechellophryne* (formerly *Sooglossus*) *pipilodryas* [49] (Figure 4). The advertisement call of *S. gardineri* has been described as ‘a single, high pitched, peep-like note’ [48] and as ‘a high-pitched squeak or whistle very similar to a cricket’ [49]. The call of *S. pipilodryas* is a similar high-pitched squeak, but unlike the call of *S. gardineri*, the call of *S. pipilodryas* is repeated six times in rapid succession. All of the sooglossids have calls with higher dominant frequencies than reported here for *Nasikabatrachus sahyadrensis* (Figure 4), but this is almost certainly due to their uniformly smaller adult body sizes (e.g. 10–44 mm) [48], [49].

One of the most unique aspects of the vocal behavior of *N. sahyadrensis* is that males called from underground. Many frogs are known to call from excavated burrows or nests in the soil [2]. Males of *N. sahyadrensis* called from below a thin surface layer of loose soil and humus at the entrance of a small tunnel, itself also partially filled with loose soil. The top layer of soil was typically thick enough to fully conceal the body of the frog from view, but not so thick as to render vocalizations inaudible or to conceal gross movements associated with calling, such as contraction of the trunk musculature and inflation of the vocal sac. Such below-the-soil-surface calling behavior appears to be relatively uncommon in frogs [2]. Two burrowing myobatrachid frogs from Western Australia, *Myobatrachus gouldii* [51] and *Arenophyrne rotunda* [52], have also been reported to call from just below the surface of the soil, though both more regularly call from above the surface. At present, we do not know what effects the behavior of calling under the soil has on the acoustic structure of vocalizations in *Nasikabatrachus sahyadrensis*. Our emphasis in this study was on capturing the males we recorded, which unfortunately, due to the tendency of these skittish animals to retreat further underground in response to slight disturbances, required rapidly digging into their calling sites. Informal comparisons between vocalizations recorded from males calling below the soil (Audio S1) with those recorded from males captured and placed above the soil (Audio S2, Audio S3, and Video S1) do not suggest any strong differences in the overall acoustic structure of calls.

Another interesting feature of vocal communication in *N. sahyadrensis* is that the animal lacks a tympanum [24], [29]. Most frogs have tympanic middle ears in which the tympanum, which sits flush with the side of the head, transmits sound energy to the inner ear via a single middle ear ossicle (the columella, or stapes) [53]. However, some frogs, for example in the genera *Bombina* (Bombinatoridae), *Atelopus* and *Mertensophryne* (Bufonidae), and *Eleutherodactylus* (Eleutherodactylidae), lack part or all of a tympanic middle ear [54]–[60]. Interestingly, all four of the known sooglossid species from the Seychelles archipelago, the closest living relatives of *Nasikabatrachus sahyadrensis*, also lack tympanic middle ears [28], [50]. In species completely lacking a tympanic middle ear, the body wall and lungs can serve the function of transmitting sound energy to the inner ear [55], [56]. Boistel et al. [50] recently described a mechanism of sound transmission to the inner ear in a sooglossid frog lacking a tympanic middle ear, *Sechellophryne gardineri*, involving bone conduction enhanced by the resonance characteristics of the mouth cavity. At present, it is not known whether *Nasikabatrachus sahyadrensis* possesses a functional middle ear for detecting sound. Given the animal's fossorial lifestyle, the use of substrate borne vibrations in hearing and communication also seems of likely importance, as shown for some other frogs [61]–[64]. Hence, *N. sahyadrensis* could serve as an important model for further comparative studies of sound and vibration sensitivity in a potentially earless animal.

In conclusion, the acoustical and statistical analyses reported here represent a first step toward better understanding the vocal communication system of a fossorial and superficially earless frog that is a relict of an early evolutionary divergence in anurans. The data reported here should facilitate future experimental work to investigate the mechanisms and function of underground calling in this species, as this is a fairly unusual behavior in frogs. It will be especially important in future studies of signal function and perception to couple acoustic and seismic recordings with playback experiments. In addition, the data and Supporting Information presented here should aid efforts to better delimit the known range of this endangered species in the Western Ghats and to determine and monitor the size and conservation status of known populations.

## Supporting Information

Audio S1 **Acoustic recording of the 30-s sequence of call groups depicted in** **Figure 2(a)** **of the main text.**
(WAV)

Audio S2 **Acoustic recording of the first 23-s segment of two males interacting while calling in captivity and depicted in the upper plot of** **Figure 3** **.**
(WAV)

Audio S3 **Acoustic recording of the second 23-s segment of two males interacting while calling in captivity and depicted in the middle plot of** **Figure 3** **.**
(WAV)

Figure S1 **Figure depicting scatterplots showing the correlations reported in** **Table 4** **that had** ***P*** **-values below α = 0.05 (** ***N*** **= 10 for all plots; larger points are used to depict multiple individuals having the same** ***x*** **and** ***y*** **values).**
(DOCX)

Video S1 **Video recording of a calling male of** ***Nasikabatrachus sahyadrensis*** **.** The video was made using a Sony HDR-XR520V video camera (60 frames/s). Prior to video recording, the male was captured, removed from beneath the soil, and induced to call using brief exposure to a female. The calls this male produced were acoustically similar to the calls produced when males called from beneath the soil surface.
(MP4)
