# Correction: Vocal Behavior of the Elusive Purple Frog of India (*Nasikabatrachus sahyadrensis*), a Fossorial Species Endemic to the Western Ghats

**DOI:** 10.1371/journal.pone.0094121

**Published:** 2014-03-28

**Authors:** The PLOS ONE Staff

**Notice of Republication**

This article was republished on February 14, 2014, due to the release of confidential or copyrighted information. Please download the article again to view the corrected version.
