# Supplementary material for: Vocal Behavior of the Elusive Purple Frog of India (Nasikabatrachus sahyadrensis), a Fossorial Species Endemic to the Western Ghats
Source: PLoS One. 2014 Feb 7;9(2):e84809. doi: 10.1371/journal.pone.0084809 (PMC3917828; doi:10.1371/journal.pone.0084809)
Supplement: Figure S1 — Figure depicting scatterplots showing the correlations reported in Table 4 that had P -values below α = 0.05 ( N = 10 for all plots; larger points are used to depict multiple individuals having the same x and y values). (DOCX) [file pone.0084809.s004.docx]

**Figure S1. Scatterplots depicting correlations with *P* < 0.05.** The correlations depicted graphically here are reported in Table 4 of the main article. Each correlation had a *P-*value below α = 0.05, but was not significant after corrections for multiple comparisons. (*N* = 10 individual means for all plots; larger points are used to depict multiple individuals having the same *x* and *y* values.)
